# Supplementary material for: The Hippo pathway transcription factors YAP and TAZ play HPV-type dependent roles in cervical cancer
Source: Nat Commun. 2024 Jul 10;15:5809. doi: 10.1038/s41467-024-49965-9 (PMC11237029; doi:10.1038/s41467-024-49965-9)

The Hippo pathway transcription factors YAP and TAZ play HPV-type dependent roles in cervical cancer

Molly R Patterson, Joseph A Cogan, Rosa Cassidy, Daisy A Theobald, Miao Wang, James A Scarth, Chinedu A Anene, Adrian Whitehouse, Ethan L Morgan and Andrew Macdonald

Supplementary Information

Supplementary Figure 1. TAZ protein is not stabilised in cervical cancer cell lines. A) Representative western blot of HeLa lysates following treatment with cycloheximide (CHX) for the indicated time points. Lysates were probed for YAP and TAZ expression. GAPDH was used as a loading control. **B)** Densitometry analysis of YAP and TAZ expression in **A)** (n=3). Error bars represent the mean +/- standard deviation of a minimum of three biological repeats. *P<0.05, **P<0.01, ***P<0.005 (two-tailed, unpaired Student's t-test). Source data are provided as a Source Data file.

Supplementary Figure 2. HPV18 E7 overexpression in HaCaT cells increases TAZ expression. A) *WWTR1*-promoter luciferase assay in HaCaT cells stably expressing HA-HPV18 E7 (n=3). **B)** RT-qPCR analysis of *WWTR1* expression in HaCaT cells stably expressing HA-HPV18 E7 (n=4). *U6* was used as a loading control. **C)** Representative western blot of HaCaT cell lysates stably expressing HA-HPV18 E7. Lysates were probed for TAZ and HPV18 E7 expression. GAPDH was used as a loading control. **D)** Representative western blot of HPV16+ CaSKi cells after transfection with HPV16 E6/E7 targeting siRNA. Cell lysates were probed for TAZ, YAP, HPV16 E6 and HPV16 E7 expression. GAPDH was used as a loading control. **E)** Densitometry analysis of TAZ expression in **D)** (n=3). Error bars represent the mean +/- standard deviation of a minimum of three biological repeats. *P<0.05, **P<0.01, ***P<0.005 (two-tailed, unpaired Student's t-test). Source data are provided as a Source Data file.

Supplementary Figure 3. HPV18 E7-mediated TAZ expression in regulated by the ERK1/2-SP1 signalling A) RT-qPCR analysis of *WWTR1* expression in SW756 cells following Mithramycin A (Mith A) treatment (n=4). *U6* was used as a loading control. **B)** RT-qPCR analysis of *WWTR1* expression in MS751 cells following Mith A treatment (n=4). *U6* was used as a loading control. **C)** Representative western blot of HeLa cells following transfection of Trunc-SP1. Cell lysates were probed for TAZ and FLAG expression. GAPDH was used as a loading control. **D)** *WWTR1*-promoter luciferase assay in HeLa cells following transfection of

Trunc-SP1 (n=3). **E)** RT-qPCR analysis of *WWTR1* expression in HeLa cells following transfection of Trunc-SP1 (n=3). *U6* was used as a loading control. **F)** RT-qPCR analysis of HPV18 *E6* or *E7* expression in HeLa cells after transfection with HPV18 E6/E7 targeting siRNA +/- SP1 2TD overexpression (n=3). *U6* was used as a loading control. Error bars represent the mean +/- standard deviation of a minimum of three biological repeats. *P<0.05, **P<0.01, ***P<0.005 (two-tailed, unpaired Student's t-test). Source data are provided as a Source Data file.

Supplementary Figure 4. Knockdown of TAZ in additional HPV18+ cervical cancer cells reduces proliferation A) RT-qPCR analysis of *WWTR1* or *YAP1* expression in TAZ KD SW756 cells (n=3). *U6* was used as a loading control. **B)** RT-qPCR analysis of *WWTR1* or *YAP1* expression in TAZ KD SW756 cells (n=3). *U6* was used as a loading control **C)** Representative western blot of TAZ KD SW756 cells. Cell lysates were probed for YAP and TAZ expression. GAPDH was used as a loading control. **D)** Representative western blot of TAZ KD MS751 cells. Cell lysates were probed for YAP and TAZ expression. GAPDH was used as a loading control **E)** Growth curve analysis of TAZ KD SW756 cells (n=3). **F)** Growth curve analysis of TAZ KD MS751 cells (n=3). **G)** Colony formation assay of TAZ KD SW756 cells (n=4). **H)** Colony formation assay of TAZ KD SW756 cells (n=3). Error bars represent the mean +/- standard deviation of a minimum of three biological repeats. *P<0.05, **P<0.01, ***P<0.005 (two-tailed, unpaired Student's t-test). Source data are provided as a Source Data file.

Supplementary Figure 5. TAZ knockdown inhibits wound healing, migration, invasion and cellular protrusions A) Microscopy analysis of wound healing assays. Confluent monolayers of TAZ KD HeLa cells were scratched with a pipette tip and imaged. Cells were then reimaged after 24 hours (images shown). Black line indicates edge of wound. **B)** Analysis

of % wound closure calculate from **A)** (n=3). **C)** Transwell migration assay of TAZ KD HeLa cells (n=3). **D)** Cell Invasion assay of TAZ KD HeLa cells (n=4). **E)** Immunofluorescence microscopy analysis of Rhodamine-Phalloidin staining (red) in TAZ KD HeLa cells. DAPI stained nuclei (blue). Scale bar 10 μm. **F)** Measurement of filopodia length in TAZ KD HeLa cells (n=10). **G)** Average number of filopodia per cell in TAZ KD HeLa cells (n=10). Error bars represent the mean +/- standard deviation of a minimum of three biological repeats. *P<0.05, **P<0.01, ***P<0.005 (two-tailed, unpaired Student's t-test). Source data are provided as a Source Data file.

Supplementary Figure 6. TAZ overexpression only increases proliferation in HPV18+ cervical cancer cells A) Representative western blots of HeLa cells transfected with FLAG-TAZ or FLAG-YAP. Cell lysates were probed for FLAG expression. GAPDH was used as a loading control. **B)** Growth curve analysis of HeLa cells transfected with FLAG-TAZ or FLAG-YAP (n=3). **C)** Colony formation assay of HeLa cells transfected with FLAG-TAZ or FLAG-YAP (n=4). **D)** Soft agar assay in HeLa cells transfected with FLAG-TAZ or FLAG-YAP (n=3). **E)** Representative western blot of SiHa cells transfected with FLAG-TAZ or FLAG-YAP. Cell lysates were probed for FLAG expression. GAPDH was used as a loading control. **F)** Growth curve analysis of SiHa cells transfected with FLAG-TAZ or FLAG-YAP (n=3). **G)** Colony formation assay of SiHa cells transfected with FLAG-TAZ or FLAG-YAP (n=3). **H)** Soft agar assay in SiHa cells transfected with FLAG-TAZ or FLAG-YAP (n=3). Error bars represent the mean +/- standard deviation of a minimum of three biological repeats. *P<0.05, **P<0.01, ***P<0.005 (two-tailed, unpaired Student's t-test). Source data are provided as a Source Data file.

Supplementary Figure 7. TAZ knockdown does not decrease canonical YAP-dependent gene expression. A) RT-qPCR analysis of *AREG* expression in TAZ, YAP or YAP/TAZ (Y/T)

KD HeLa cells (n=3). *U6* was used as a loading control. **B)** RT-qPCR analysis of *CCND1* expression in TAZ, YAP or YAP/TAZ (Y/T) KD HeLa cells (n=3). *U6* was used as a loading control. **C)** RT-qPCR analysis of *CD133* expression in TAZ, YAP or YAP/ TAZ (Y/T) KD HeLa cells (n=3). *U6* was used as a loading control. **D)** RT-qPCR analysis of *CYR61* expression in TAZ, YAP or YAP/ TAZ (Y/T) KD HeLa cells (n=3). *U6* was used as a loading control. Error bars represent the mean +/- standard deviation of a minimum of three biological repeats. *P<0.05, **P<0.01, ***P<0.005 (two-tailed, unpaired Student's t-test). Source data are provided as a Source Data file.

Supplementary Figure 8. *TOGARAM2* is a TAZ dependent gene. A) RT-qPCR analysis of *TOGARAM2* expression in TAZ, YAP or YAP/TAZ (Y/T) KD HeLa cells (n=3). *U6* was used as a loading control. **B)** RT-qPCR analysis of *TOGARAM2* expression in TAZ KD SW756 cells (n=3). *U6* was used as a loading control. **C)** RT-qPCR analysis of TOGARAM2 expression in TAZ KD MS751 cells (n=3). *U6* was used as a loading control. **D)** RT-qPCR analysis of *TOGARAM2* expression in HeLa cells following either DMSO, 6079510 or Ivermectin treatment (n=3). *U6* was used as a loading control. **E)** RT-qPCR analysis of *TOGARAM2* expression in C33A cells stably expressing HA-HPV18 E7 plus 6079510 treatment (n=3). *U6* was used as a loading control. **F)** RT-qPCR analysis of *TOGARAM2* expression in HeLa cells following DMSO or verteporfin treatment (n=4). *U6* was used as a loading control. Error bars represent the mean +/- standard deviation of a minimum of three biological repeats. *P<0.05, **P<0.01, ***P<0.005 (two-tailed, unpaired Student's t-test). Source data are provided as a Source Data file.

Supplementary Figure 9. TOGARAM2 overexpression increases proliferation of cervical cancer cells. A) Representative western blots of HeLa cells transfected with FLAG-TOGARAM2. Cell lysates were probed for FLAG expression. GAPDH was used as a loading

control. **B)** Growth curve analysis of HeLa cells transfected with FLAG-TOGARAM2 (n=3). **C)** Colony formation assay of HeLa cells transfected with FLAG-TOGARAM2 (n=3). **D)** Soft agar assay in HeLa cells transfected with FLAG-TOGARAM2 (n=3). **E)** Growth curve analysis of C33A cells transfected with FLAG-TOGARAM2 (n=3). **F)** Colony formation assay of C33A cells transfected with FLAG-TOGARAM2 (n=3). **G)** Soft agar assay in C33A cells transfected with FLAG-TOGARAM2 (n=3). **H)** Transwell migration assay of C33A cells transfected with FLAG-TOGARAM2 (n=3). **I)** Transwell Invasion assay of C33A cells transfected with FLAG-TOGARAM2 (n=4). Error bars represent the mean +/- standard deviation of a minimum of three biological repeats. *P<0.05, **P<0.01, ***P<0.005 (two-tailed, unpaired Student's t-test). Source data are provided as a Source Data file.

Supplementary Figure 10. TOGARAM2 promotes proliferation in HPV18+, but not HPV16+, cervical cancer cell lines. A) RT-qPCR analysis of *TOGARAM2* expression in TOGARAM2 KD SiHa cells (n=3). *U6* was used as a loading control. **B)** Growth curve analysis of TOGARAM2 KD SiHa cells. **C)** RT-qPCR analysis of *TOGARAM2* expression in TOGARAM2 KD SW756 cells (n=3). *U6* was used as a loading control. **D)** Growth curve analysis of TOGARAM2 KD SW756 cells (n=3). Error bars represent the mean +/- standard deviation of a minimum of three biological repeats. *P<0.05, **P<0.01, ***P<0.005 (two-tailed, unpaired Student's t-test). Source data are provided as a Source Data file.

Supplementary Data 1 List of primer sequences used in this study.

Supplementary Data 2- Upregulated DEGs in TAZ KD HeLa cells.

Supplementary Data 3- Gene Ontology analysis of genes upregulated in TAZ KD HeLa cells. Terms with adjusted P<0.05 (ClusterProfiler Over-representation test) are highlighted green.

Supplementary Data 4- Downregulated DEGs in TAZ KD HeLa cells.

Supp Figure 1

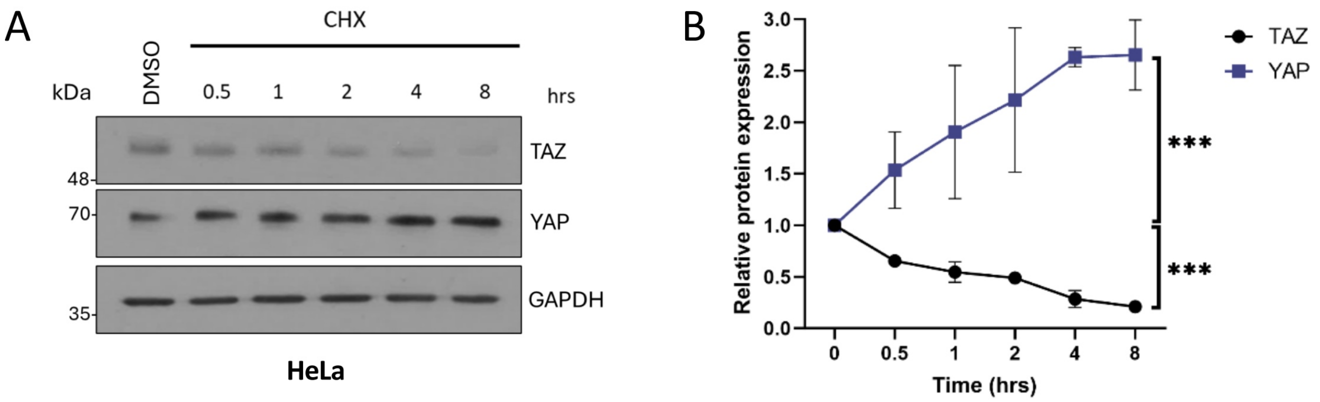

Supp Figure 2

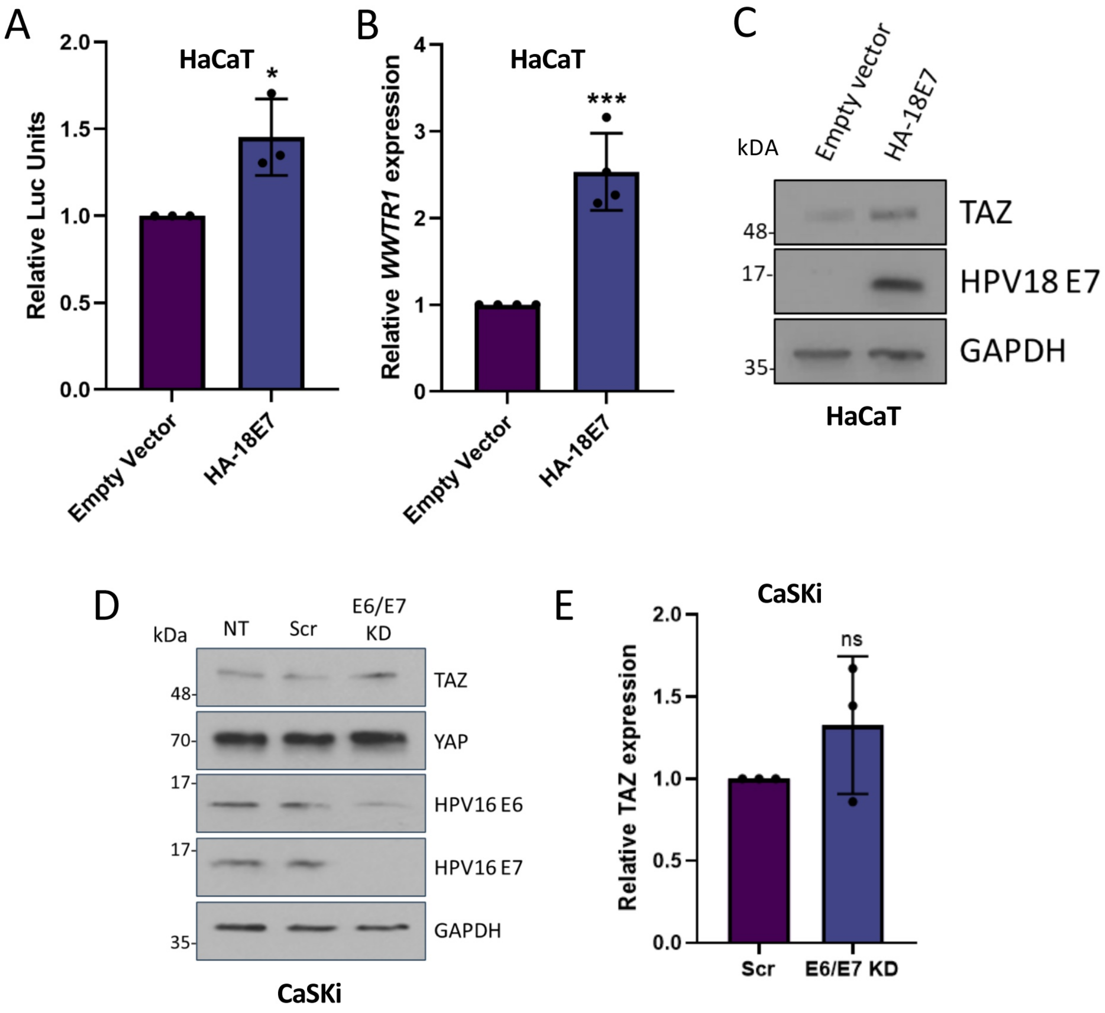

Supp Figure 3

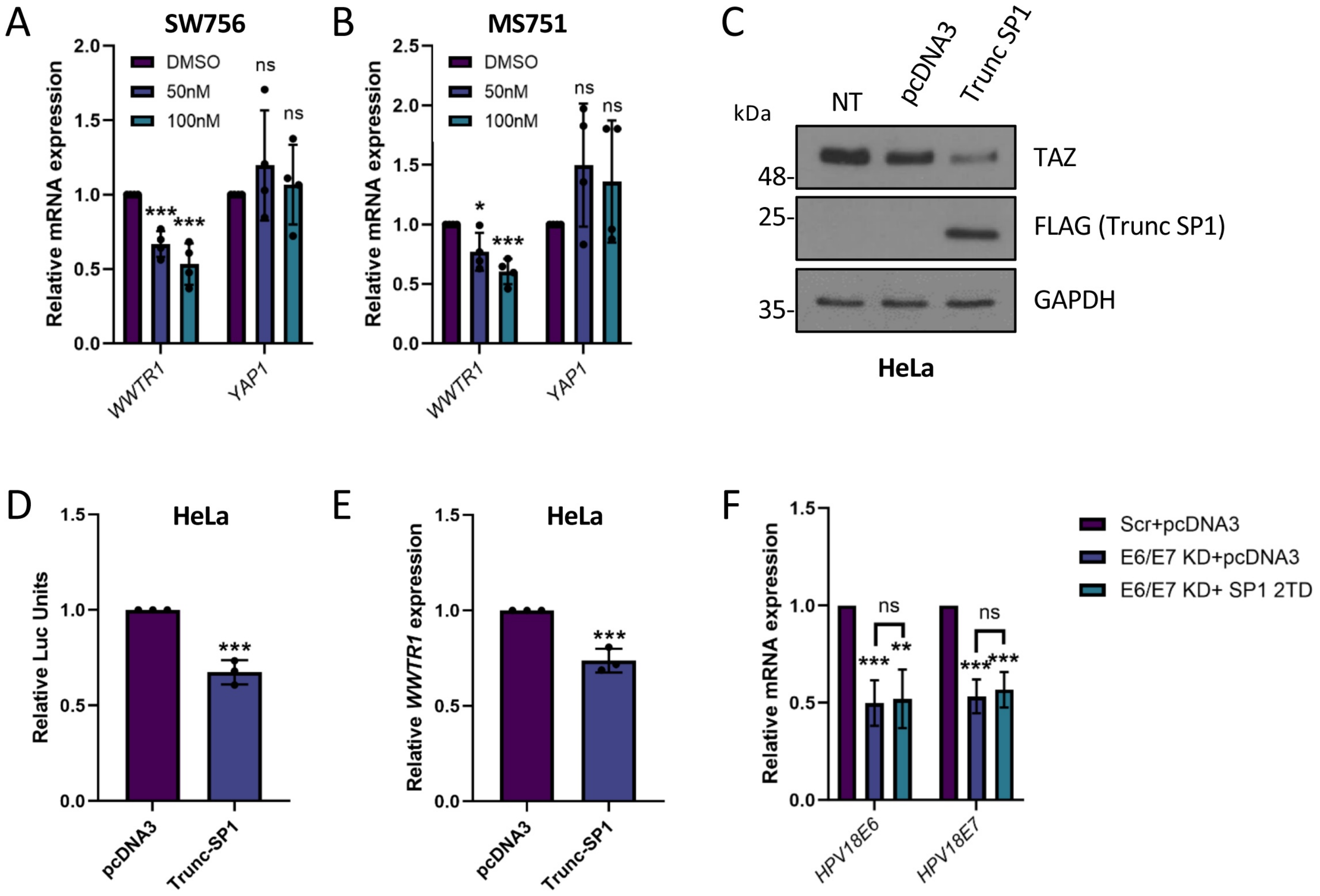

Supp Figure 4

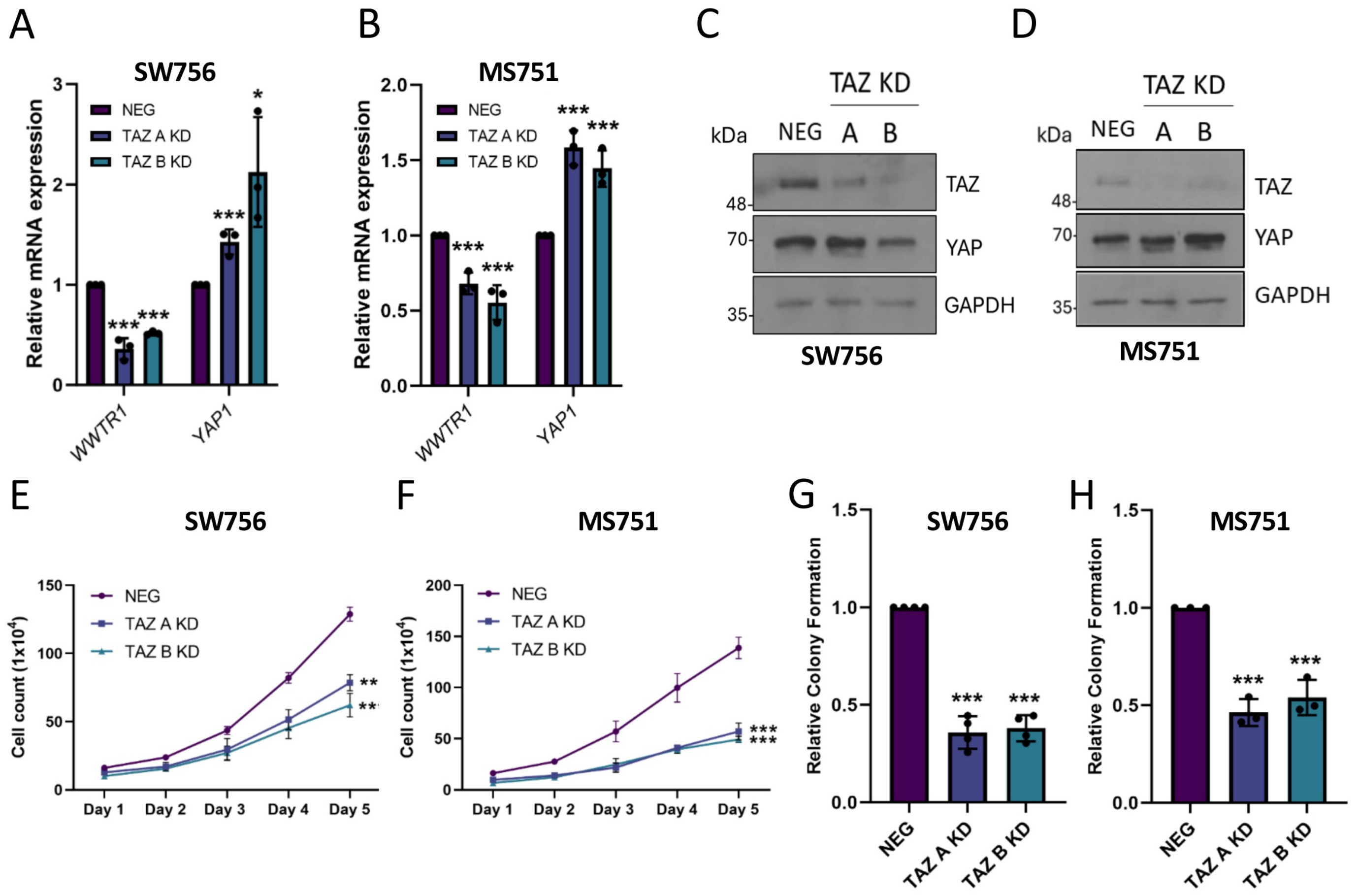

Supp Figure 5

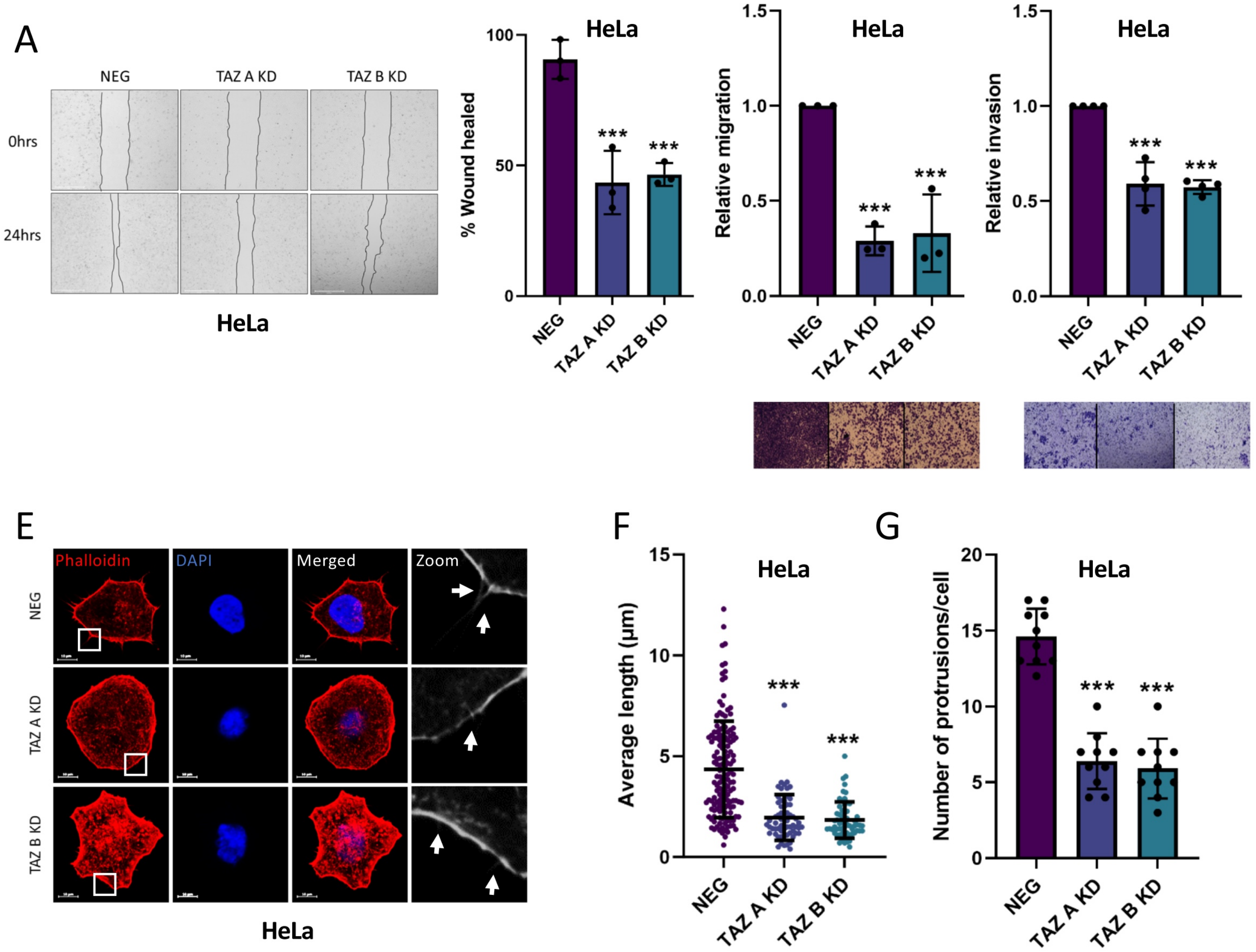

Supp Figure 6

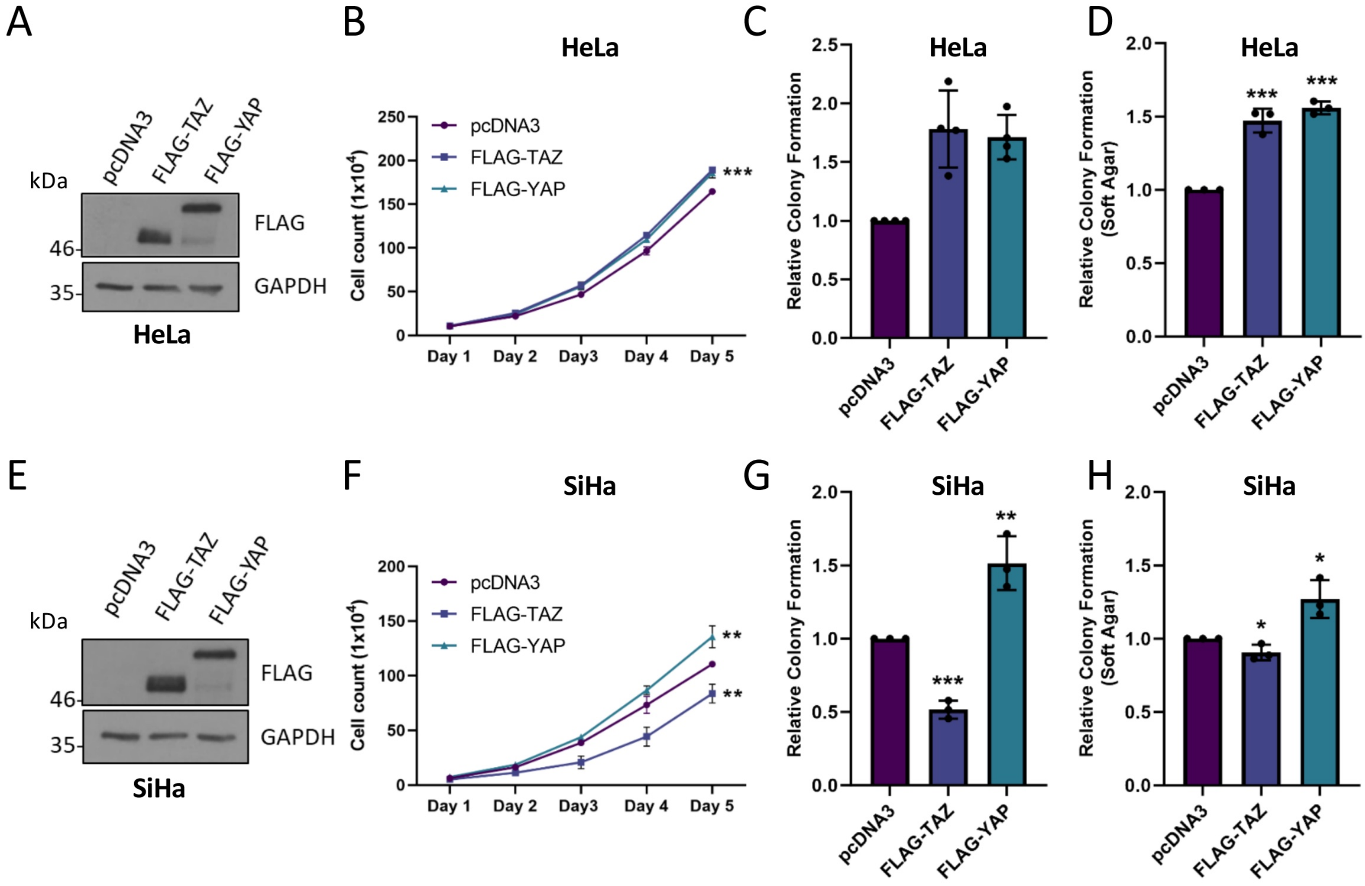

Supp Figure 7

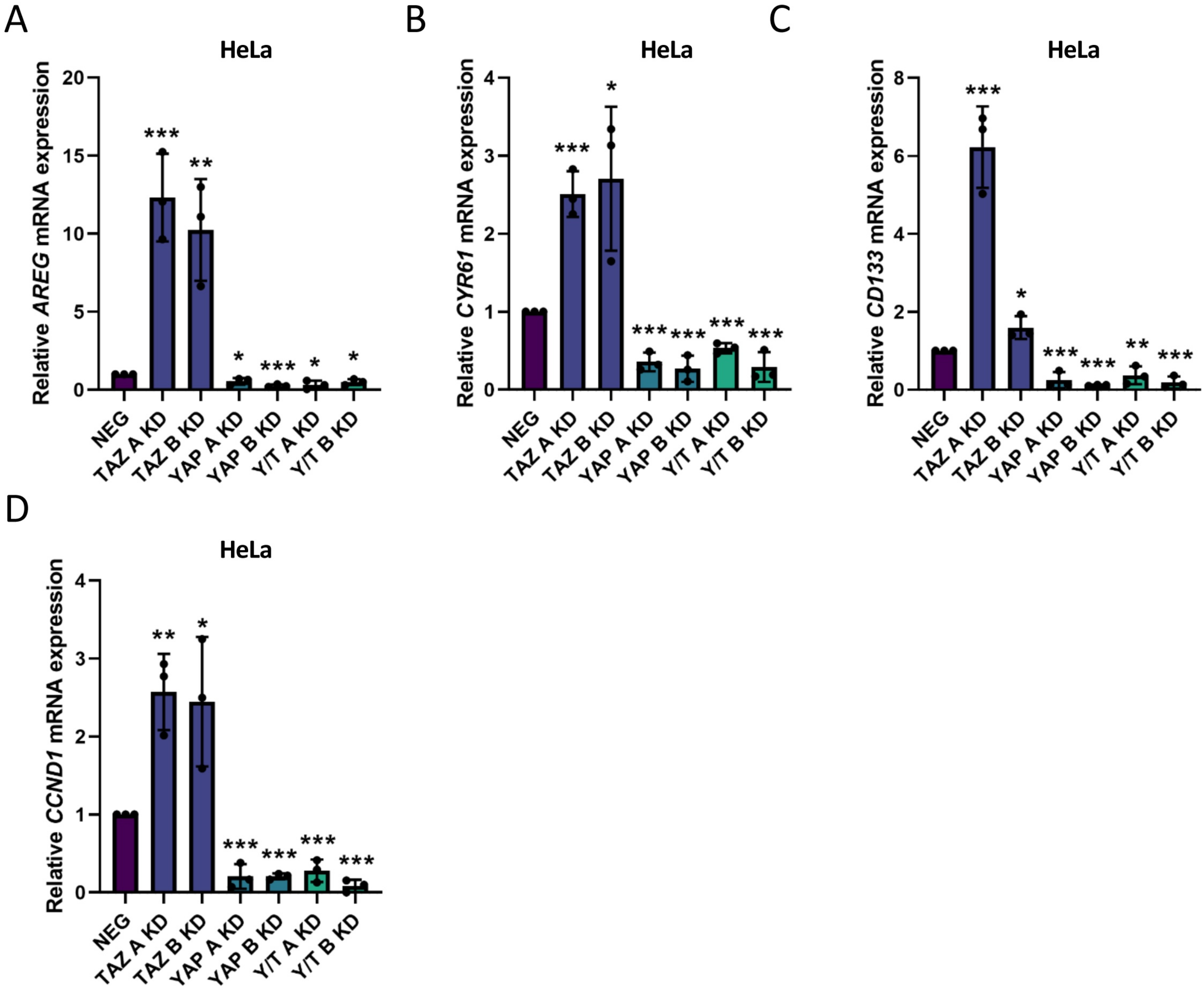

Supp Figure 8

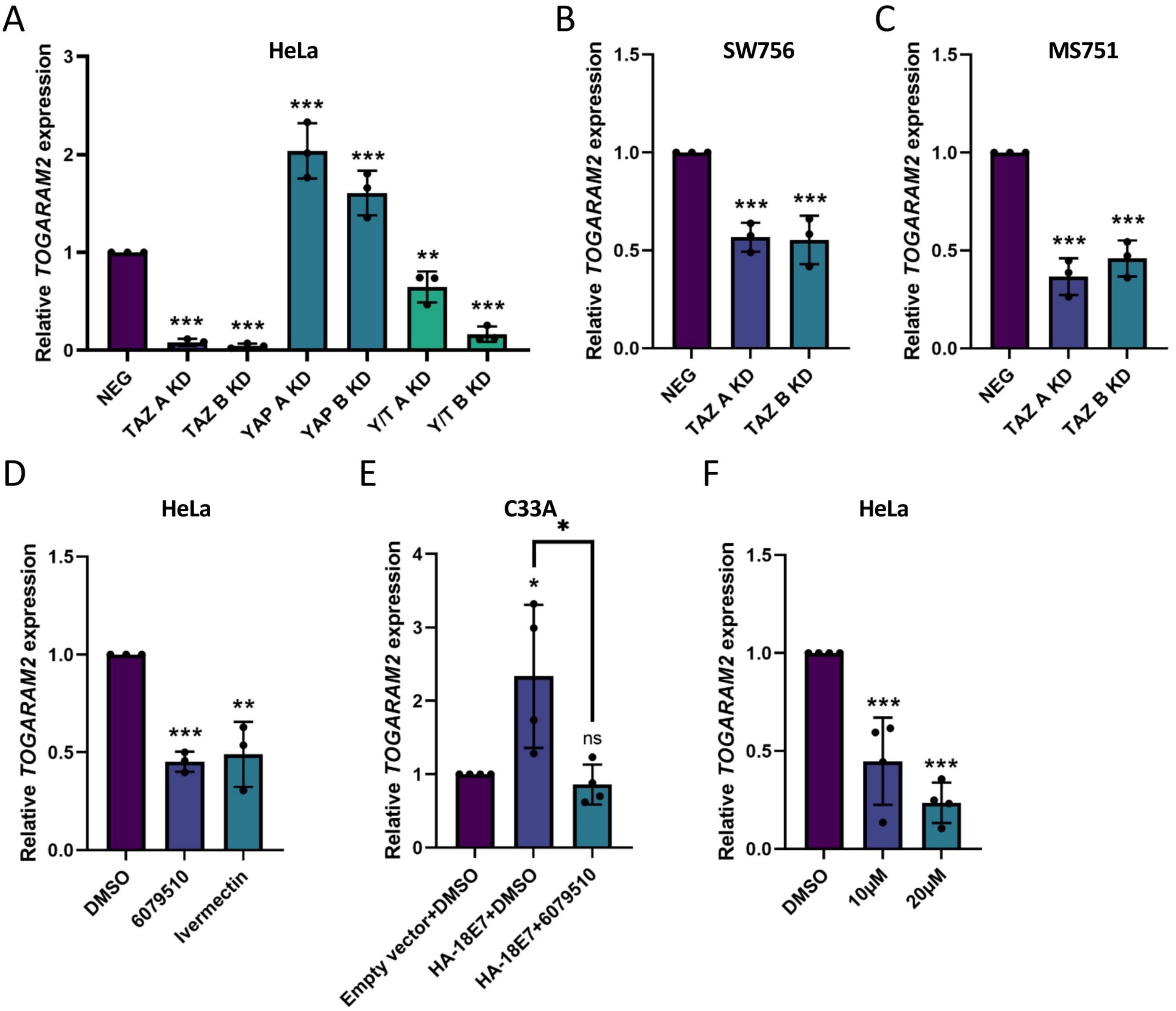

Supp Figure 9

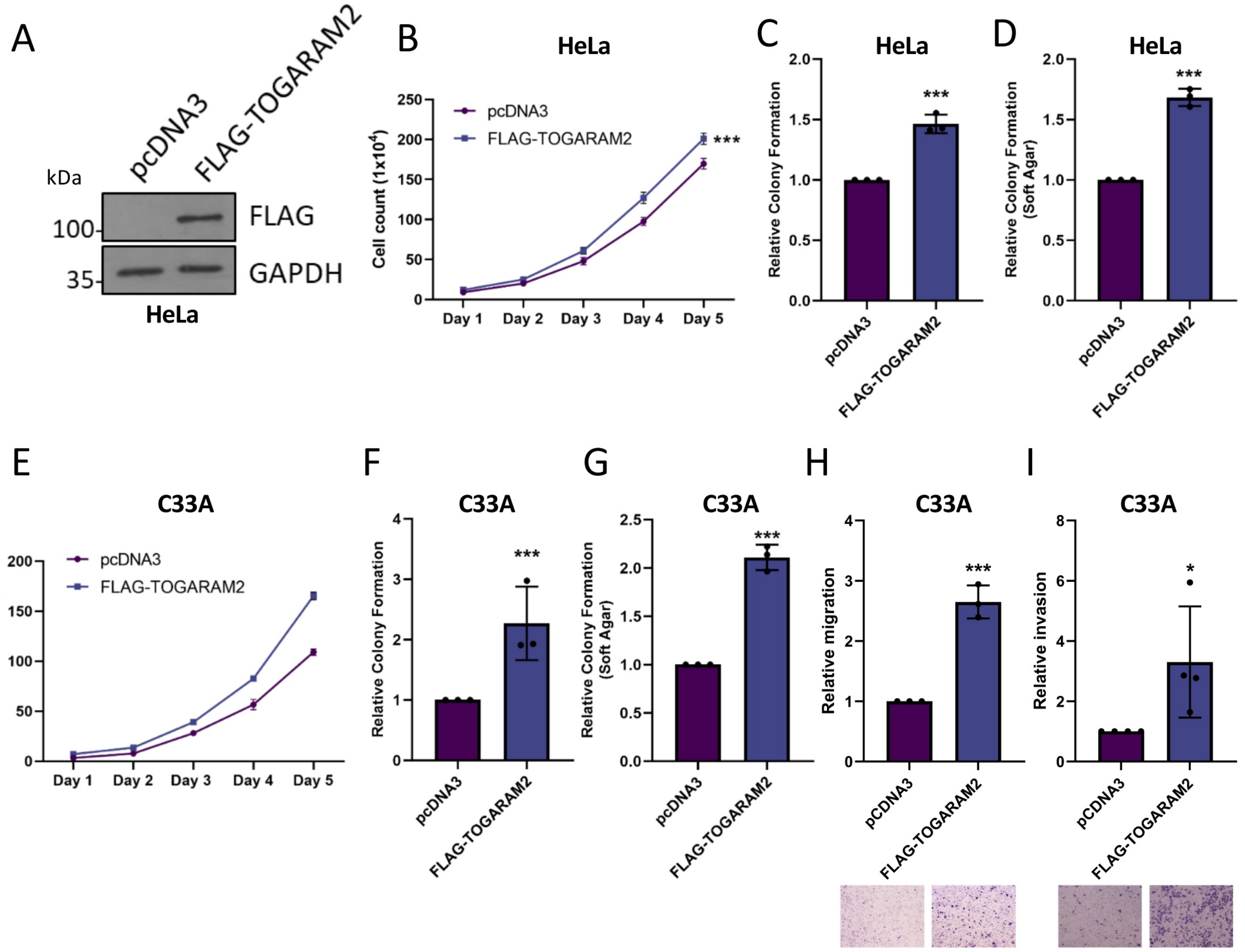

Supp Figure 10

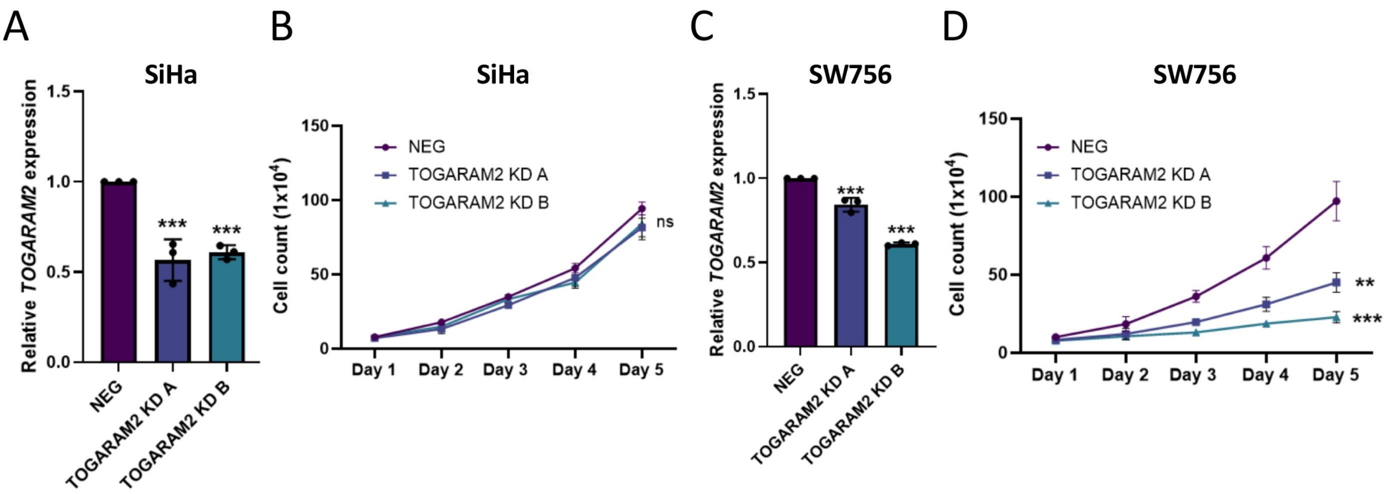

Supplement: Supplementary file 1 — Supplementary Information [file 41467_2024_49965_MOESM1_ESM.pdf]
