# Supplementary material for: The Hippo pathway transcription factors YAP and TAZ play HPV-type dependent roles in cervical cancer
Source: Nat Commun. 2024 Jul 10;15:5809. doi: 10.1038/s41467-024-49965-9 (PMC11237029; doi:10.1038/s41467-024-49965-9)
Supplement: Supplementary file 3 — Description of Additional Supplementary Files [file 41467_2024_49965_MOESM3_ESM.pdf]

Description of Additional Supplementary Files

File Name: Supplementary Data S1-S4
Description: Tables with RNA-sequencing data and lists of primers used in the experiments
