# Supplementary material for: The Hippo pathway transcription factors YAP and TAZ play HPV-type dependent roles in cervical cancer
Source: Nat Commun. 2024 Jul 10;15:5809. doi: 10.1038/s41467-024-49965-9 (PMC11237029; doi:10.1038/s41467-024-49965-9)
Supplement: Supplementary file 5 — Reporting Summary [file 41467_2024_49965_MOESM5_ESM.pdf]

nature portfolio

Corresponding author(s): Andrew Macdonald
Molly Patterson

Last updated by author(s): 25/03/2024

Reporting Summary

Nature Portfolio wishes to improve the reproducibility of the work that we publish. This form provides structure for consistency and transparency in reporting. For further information on Nature Portfolio policies, see our Editorial Policies and the Editorial Policy Checklist.

Statistics

For all statistical analyses, confirm that the following items are present in the figure legend, table legend, main text, or Methods section.

n/a	Confirmed	
☐	☒	The exact sample size (*n*) for each experimental group/condition, given as a discrete number and unit of measurement
☐	☒	A statement on whether measurements were taken from distinct samples or whether the same sample was measured repeatedly
☐	☒	The statistical test(s) used AND whether they are one- or two-sided<br>*Only common tests should be described solely by name; describe more complex techniques in the Methods section.*
☐	☒	A description of all covariates tested
☐	☒	A description of any assumptions or corrections, such as tests of normality and adjustment for multiple comparisons
☐	☒	A full description of the statistical parameters including central tendency (e.g. means) or other basic estimates (e.g. regression coefficient) AND variation (e.g. standard deviation) or associated estimates of uncertainty (e.g. confidence intervals)
☐	☒	For null hypothesis testing, the test statistic (e.g. *F*, *t*, *r*) with confidence intervals, effect sizes, degrees of freedom and *P* value noted<br>*Give P values as exact values whenever suitable.*
☒	☐	For Bayesian analysis, information on the choice of priors and Markov chain Monte Carlo settings
☒	☐	For hierarchical and complex designs, identification of the appropriate level for tests and full reporting of outcomes
☐	☒	Estimates of effect sizes (e.g. Cohen's *d*, Pearson's *r*), indicating how they were calculated

Our web collection on statistics for biologists contains articles on many of the points above.

Software and code

Policy information about availability of computer code

Data collection	RT-qPCR was performed on a CFX Connect Real-Time PCR Detection System (Bio-Rad). For RNA-Sequencing, the NovaSeq 6000 (PE150) platform was used.
Data analysis	Confocal imaging data was quantified in ImageJ. All graphs and statistical analyses were generated in GraphPad Prism version9. Adaptors trimmed and read quality filtered using Trimmomatic (V 0.39). Reads aligned to human genome (Hg38) using HISAT2 (V 2.1.0). Counts generated using HTSeq (V 0.11.1). Differential expression analysis was performed using R package EdgeR. No custom algorithms utilised.

For manuscripts utilizing custom algorithms or software that are central to the research but not yet described in published literature, software must be made available to editors and reviewers. We strongly encourage code deposition in a community repository (e.g. GitHub). See the Nature Portfolio guidelines for submitting code & software for further information.

Data

Policy information about availability of data

All manuscripts must include a data availability statement. This statement should provide the following information, where applicable:

- Accession codes, unique identifiers, or web links for publicly available datasets
- A description of any restrictions on data availability
- For clinical datasets or third party data, please ensure that the statement adheres to our policy

Source data have been provided in the Source Data file. The RNAseq data generated in this study have been deposited in the GEO database under accession code

GSE261673. The analyzed RNAseq data were available in Supplementary Information files. The TCGA data were accessed and are available through cBioPortal. The remaining data were provided with this paper within the Article, Supplementary Information or Source Data files. Source data are provided with this paper.

Research involving human participants, their data, or biological material

Policy information about studies with human participants or human data. See also policy information about sex, gender (identity/presentation), and sexual orientation and race, ethnicity and racism.

Reporting on sex and gender	Patient cytology samples used in all studies were de-identified prior to transfer to the research laboratory and thus the distribution of gender is unknown. All samples were cervical tissue which by definition originate only from female patients.
Reporting on race, ethnicity, or other socially relevant groupings	These are unknown
Population characteristics	See above, these are also unknown
Recruitment	Patients were undergoing clinically indicated procedures that involved removal of cytology samples. Excess sample was provided to support the study.
Ethics oversight	The East of Scotland Research Ethics Service has given generic approval to the Scottish HPV Archive as a Research Tissue Bank (REC Ref 11/AL/0174) for HPV related research on anonymised archive samples. Samples are available for the present project through application to the Archive Steering Committee (HPV Archive Application Ref 0034).

Note that full information on the approval of the study protocol must also be provided in the manuscript.

Field-specific reporting

Please select the one below that is the best fit for your research. If you are not sure, read the appropriate sections before making your selection.

☒ Life sciences ☐ Behavioural & social sciences ☐ Ecological, evolutionary & environmental sciences

For a reference copy of the document with all sections, see nature.com/documents/nr-reporting-summary-flat.pdf

Life sciences study design

All studies must disclose on these points even when the disclosure is negative.

Sample size	Because HPV18 positive SCC is relatively rare all available samples were included in the study. For experiments in cell lines no sample size calculation was conducted. We selected samples sizes of sufficient size to ensure reproducibility of our findings as well as large enough to perform statistical analyses (at least n=3 unless stated otherwise). Our rational for selecting this sample size was primarily based on previous experience with these assays informing us on the number of replicates required to achieve statistical significance given the variance of each assay.
Data exclusions	No data were excluded from this study. We pre-established our exclusion criteria as a rejection of a dataset if either our positive controls or negative controls failed.
Replication	We repeated experiments at least three times or the number indicated in the figure legends.
Randomization	Experiments were not randomized. Data variability was controlled through the inclusion of multiple biological replicates and inclusion of multiple technical replicates within an experiment.
Blinding	Blinding was not appropriate for the nature of this study. The study does not involve clinical research.

Reporting for specific materials, systems and methods

We require information from authors about some types of materials, experimental systems and methods used in many studies. Here, indicate whether each material, system or method listed is relevant to your study. If you are not sure if a list item applies to your research, read the appropriate section before selecting a response.

Materials & experimental systems

n/a	Involved in the study
☐	☒ Antibodies
☐	☒ Eukaryotic cell lines
☒	☐ Palaeontology and archaeology
☒	☐ Animals and other organisms
☒	☐ Clinical data
☒	☐ Dual use research of concern
☒	☐ Plants

Methods

n/a	Involved in the study
☒	☐ ChIP-seq
☒	☐ Flow cytometry
☒	☐ MRI-based neuroimaging

Antibodies

Antibodies used

TAZ- BD Pharmingen; 1:500; Cat No: 560235; https://www.bdbiosciences.com/en-gb/products/reagents/microscopy-imaging-reagents/immunofluorescence-reagents/purified-mouse-anti-taz.560235
YAP- Cell Signalling Technology; 1:1000; Cat No: 14074; https://www.cellsignal.com/products/primary-antibodies/yap-d8h1x-xp-174-rabbit-mab/14074
HPV16 E7- Santa Cruz Biotechnology; 1:250; Cat No: sc-1587; https://www.scbt.com/de/p/hpv16-e7-antibody-c-20
HPV18 E7- Abcam; 1:1000; Cat No: ab100953; https://www.abcam.com/products/primary-antibodies/hpv18-e7-antibody-8e2-ab100953.html
HPV16 E6- GeneTex Inc; 1:500; Cat No: GTX132686; https://www.genetex.com/Product/Detail/Human-Papillomavirus-type-16-E6-antibody/GTX132686
HPV18 E6- Santa Cruz Biotechnology; 1:500; Cat No: sc-365089; https://www.scbt.com/p/hpv18-e6-antibody-g-7
GFP- Santa Cruz Biotechnology; 1:2500; Cat No: sc-9996; https://www.scbt.com/p/gfp-antibody-b-2
Phospho-ERK1/2 (T202/Y204)- Cell Signalling Technology; 1:1000; Cat No: 9101; https://www.cellsignal.com/products/primary-antibodies/phospho-p44-42-mapk-erk1-2-thr202-tyr204-antibody/9101
ERK1/2- Cell Signalling Technology; 1:1000; Cat No: 9102; https://www.cellsignal.com/products/primary-antibodies/p44-42-mapk-erk1-2-antibody/9102?gclid=EAIaIQobChMI4Y2H7Ir-hAMVnJpQBh2oOQ-7EAAYAiAAEgLFXvD_BwE&gclsrc=aw.ds
FLAG- Sigma-Aldrich; 1:1000; Cat No: F1804; https://www.sigmaaldrich.com/GB/en/product/sigma/f1804?utm_source=google&utm_medium=cpc&utm_campaign=15001183107&utm_content=127306761543&gclid=EAIaIQobChMIw5eyp47-hAMVW5NQBh0njQ4gEAAYAiAAEgLCbfD_BwE
GAPDH- Santa Cruz Biotechnology; 1:5000; Cat No: sc-365062; https://www.scbt.com/p/gapdh-antibody-g-9

Validation

Only antibodies that validated according to the manufacturer's instructions or utilised in previously published articles were used in this study. Antibodies such as E6/E7 were validated using lysates from both positive and negative controls (e.g. overexpression samples or knockdown samples), others such as P-ERK1/2 were tested with small molecule inhibitors e.g. UO126.

Eukaryotic cell lines

Policy information about cell lines and Sex and Gender in Research

Cell line source(s)

All cell lines obtained from the ATCC
HaCaT (immortalized human keratinocytes)
C33A (HPV negative cervical squamous carcinoma cells)
SiHa (HPV16+ cervical squamous carcinoma cells)
CaSKi (HPV16+ cervical squamous carcinoma cells),
SW756 (HPV18+ cervical squamous carcinoma cells),
C4-I (HPV18+ squamous carcinoma of the uterine cervix),
HeLa (HPV18+ cervical epithelial adenocarcinoma),
MS751 (HPV45+ cervical squamous cell carcinoma derived from lymph node)
HEK293T (human embryonic kidney cell expressing SV40 T antigen) used for generating retroviral vectors.

Authentication

STR profiling of all cell lines

Mycoplasma contamination

Cells were negative for mycoplasma by PCR and gel electrophoresis

Commonly misidentified lines
(See ICLAC register)

None of the cell lines in this study is found in the ICLAC register.

Plants

Seed stocks	*Report on the source of all seed stocks or other plant material used. If applicable, state the seed stock centre and catalogue number. If plant specimens were collected from the field, describe the collection location, date and sampling procedures.*
Novel plant genotypes	*Describe the methods by which all novel plant genotypes were produced. This includes those generated by transgenic approaches, gene editing, chemical/radiation-based mutagenesis and hybridization. For transgenic lines, describe the transformation method, the number of independent lines analyzed and the generation upon which experiments were performed. For gene-edited lines, describe the editor used, the endogenous sequence targeted for editing, the targeting guide RNA sequence (if applicable) and how the editor was applied.*
Authentication	*Describe any authentication procedures for each seed stock used or novel genotype generated. Describe any experiments used to assess the effect of a mutation and, where applicable, how potential secondary effects (e.g. second site T-DNA insertions, mosiacism, off-target gene editing) were examined.*
